# The Effect of Dual Sensory Impairment and Multimorbidity Patterns on Functional Impairment: A Longitudinal Cohort of Middle-Aged and Older Adults in China

**DOI:** 10.3389/fnagi.2022.807383

**Published:** 2022-04-08

**Authors:** Qiong Wang, Shimin Zhang, Yi Wang, Dan Zhao, Xi Chen, Chengchao Zhou

**Affiliations:** ^1^Centre for Health Management and Policy Research, School of Public Health, Cheeloo College of Medicine, Shandong University, Jinan, China; ^2^NHC Key Lab of Health Economics and Policy Research, Shandong University, Jinan, China; ^3^Department of Health Policy and Management, Yale School of Public Health, New Haven, CT, United States; ^4^Department of Economics, Yale University, New Haven, CT, United States

**Keywords:** dual sensory impairment, multimorbidity, functional impairment, longitudinal population-based study, personalized medicine

## Abstract

**Objective:**

There is an urgent need to evaluate the contribution of several co-existing diseases on health. This study aims to explore the combined effect of dual sensory impairment (DSI) and multimorbidity patterns on functional impairment among middle-aged and older adults in China.

**Methods:**

Data were from 10,217 adults aged 45 or older from four waves of the China Health and Retirement Longitudinal Study (CHARLS). Sensory impairments were self-reported measures. Multimorbidity patterns were identified by using k-means cluster analyses. Functional impairment was defined using activities of daily living (ADL) scale and instrumental activities of daily living (IADL) scale. Generalized estimating equation models were estimated to assess the effect of co-occurring DSI and multimorbidity on functional impairment.

**Results:**

DSI prevalence was 50.4%, and multimorbidity prevalence was 37.7% at the baseline. The simultaneous presence of DSI and multimorbidity was associated with increased odds of ADL limitations (OR = 2.27, 95% CI: 2.11–2.43) and IADL limitations (OR = 1.89, 95% CI: 1.77–2.02). Five multimorbidity patterns were identified: the cardio-cerebrovascular pattern, the stomach-arthritis pattern, the respiratory pattern, the hepatorenal pattern, and the unspecified pattern. Compared to DSI only, DSI plus the hepatorenal pattern was most strongly associated with functional impairment (for ADL: OR = 2.70, 95% CI: 2.34–3.12; for IADL: OR = 2.04, 95% CI: 1.77–2.36).

**Conclusion:**

Middle-aged and older adults with co-occurrence of DSI and multimorbidity are at increased risk of functional impairment, especially those with multimorbidity characterized by the hepatorenal pattern. These findings imply that integrated care for DSI and multimorbidity may be a potent pathway in improving functional status.

## Introduction

Dual sensory impairment (DSI) and multimorbidity are highly prevalent among older adults and are increasing amid rapid population aging (Salive, 2013; Heine and Browning, 2014). DSI, the combination of hearing impairment (HI) and vision impairment (VI), increase the risk for costly health outcome of functional impairment (Cimarolli and Jopp, 2014; Guthrie et al., 2016; Davidson and Guthrie, 2019), with even greater impact on health outcomes than having HI only or VI only (Keller et al., 1999). Multimorbidity, defined as having two or more co-occurring chronic diseases, is also a risk factor for functional impairment (Salive, 2013; Bowling et al., 2019). The COVID-19 pandemic has emphasized to people the vital need to prevent multimorbidity, protect older adults and improve their health span (Barzilai et al., 2020). Sensory impairment (SI) and multimorbidity frequently co-occur and interrelate (Ho et al., 2020; Zheng et al., 2020; Rausch et al., 2021). Individuals with SI, especially when combined with persistent multimorbidity, may place older adults at a higher risk of functional impairment. Nonetheless, few studies have investigated the effect of co-occurring DSI and multimorbidity on functional impairment among older adults.

China has undergone dramatic demographic changes characterized by population aging, declined fertility, and increasing disease burden (Yang and Gu, 2020). Chinese older adults aged 60 + will grow to 478 million by 2050, accounting for nearly one third of the total Chinese population (Jing et al., 2020). The ability to function and move around independently is a high priority for older adults (Mueller-Schotte et al., 2019). Functional impairment is a key predictor of health outcomes including disability, hospitalization, and death (Saadeh et al., 2020), and its prevention deserves a public health priority. Interestingly, SI directly affects an older person’s ability to carry out routine activities and could be mitigated by assistive devices, it is therefore a potential target to preserve autonomy in older adults (Hochberg et al., 2012; Bouscaren et al., 2019). Identifying the effect of SI on functional impairment among older adults and using this information to better inform prevention programs may help to keep older adults in good health in later life. However, of the limited studies on the relationship between SI and functional impairment, the majority are cross-sectional (Keller et al., 1999; Cimarolli and Jopp, 2014; Guthrie et al., 2016; Davidson and Guthrie, 2019) and conducted in developed countries (Keller et al., 1999; Cimarolli and Jopp, 2014; Guthrie et al., 2016; Davidson and Guthrie, 2019; Mueller-Schotte et al., 2019). Little is known about their temporal relationship in developing countries including China.

It is estimated that among Chinese older adults, over 50% are living with DSI and 60% with multimorbidity (Heine et al., 2019; Zhao et al., 2020). DSI and multimorbidity are likely to increase rapidly and will affect a rapidly growing ageing population due to rising life-expectancy and declined fertility rate (Salive, 2013; Pinto et al., 2014). Few studies have examined the effects of multimorbidity patterns on functional impairment (Marengoni et al., 2020, 2021). An enhanced understanding of how DSI and multimorbidity patterns influence functional status could be particularly important to older adults to develop exact measures and prevent functional impairment. Additionally, multimorbidity often arises in midlife (Barnett et al., 2012; Bowling et al., 2019), and SI is also common in middle-aged adults (Rong et al., 2020). Therefore, this study aims to explore the effect of DSI and its combined effect with different multimorbidity patterns on functional impairment among Chinese middle-aged and older adults by using panel data from a nationally representative survey.

## Materials and Methods

### Participants

The data used in this study were from four waves (2011, 2013, 2015, and 2018) of the China Health and Retirement Longitudinal Study (CHARLS), a nationally representative longitudinal study that surveys about aging and health for Chinese residents aged 45 years or above (Zhao et al., 2014). The sampling method and survey contents have been reported in many studies (Rong et al., 2020; Xie et al., 2021). After excluding respondents with missing data on main variables, a total of 10,217 adults aged 45 years or above were included in our study. Selected variables in our study include sociodemographic characteristics, lifestyle factors, SI, number of chronic diseases, and functional impairment. We used the data from four waves to explore the effect of DSI and multimorbidity on functional impairment. Then, we used baseline DSI plus multimorbidity patterns to predict functional impairment in the follow-up period.

### Measures

#### Functional Impairment

Functional impairment includes limitations in activities of daily living (ADL) and instrumental activities of daily living (IADL). ADL was measured by using the activities of daily living scale (ADLs), which includes six items: dressing, eating, bathing or showering, getting in or out of bed, toileting, and controlling urination and defecation (Katz et al., 1963). IADL was measured by using the instrumental activities of daily living scale (IADLs) which includes five items: namely doing housework, cooking, shopping, managing money, and taking medication (Lawton and Brody, 1969). Each item is rated on a 4-point scale (1 = activity can be performed without any help; 4 = completely unable to perform activity without help). Participants who need help to perform at least one of these ADLs and IADLs was classified as “ADL limitations” and “IADL limitations”, respectively (Fujimoto et al., 2020). Therefore, the cut-off scores for ADLs and IADLs were 6 and 5 in this study. When respondents report one or more limitations, they were classified as having functional impairment.

#### Sensory Impairments

Hearing impairment was assessed by using one question: “Is your hearing excellent, very good, good, fair, poor? (with a hearing aid if you normally use it and without if you normally don’t).” A response of fair or poor in this question was categorized as HI. Two questions were used to assess VI: (1) “How good is your vision for seeing things at a distance (with glasses or corrective lenses), like recognizing a friend from across the street?” and (2) “How good is your vision for seeing things up close (with glasses or corrective lenses), like reading ordinary newspaper print?” We identified respondents as having VI if they reported fair or poor vision (for either long distance or near vision). Participants were categorized into four groups according to hearing and vision assessment results: no SI, HI only, VI only, and DSI.

#### Multimorbidity

CHARLS collected prevalences of 14 common chronic diseases by asking participants if they have been diagnosed with the 14 medical conditions by a doctor. In this study, we used these 14 self-reported chronic conditions to measure multimorbidity: hypertension; diabetes or high blood sugar; dyslipidemia; heart disease; cancer; chronic lung disease; liver disease; stroke; kidney disease; digestive disease; emotional, nervous, or psychiatric disease; memory-related disease; arthritis or rheumatism; and asthma. When respondents have ever been diagnosed with two or more kinds of these chronic diseases, they were regarded as having multimorbidity.

#### Covariates

Several potential confounding variables were considered in this study, including sociodemographic characteristics, lifestyle factors, and depressive symptoms (yes/no). Sociodemographic characteristics included in the analysis were as follows: age (Mean ± Standard Deviation, M ± SD), gender (male, female), educational level (lower than primary school, primary school, middle school or above), and marital status [married, unmarried (separate/divorced/widowed)], and residence (rural areas/urban areas). Lifestyle factors included smoking (never and current smoker), drinking (never and current drinker). Depressive symptoms was assessed by using the 10-item Center for Epidemiological Studies–Depression scale (CES-D 10). A cut-off score of ≥10 was used to determine the participants with depressive symptoms (Qian et al., 2017).

#### Statistical Analysis

First, baseline characteristics across five sensory impairment groups were summarized using means (SD) for continuous variables and frequencies (percentages) for categorized variables. We compared sociodemographic characteristics and lifestyle factors across all sensory impairment groups using chi-squared tests or one-way analysis of variance. Second, participants with at least two chronic diseases were included to identify multimorbidity patterns. The k-means algorithm with random initial centroids was employed to generate clusters of individuals. To obtain the optimal cluster number, the independent clustering was repeated 100 times to generate an average final solution. Participants were classified to the cluster in which they had the highest membership probability. To name multimorbidity patterns, observed/expected (O/E) ratios and disease exclusivity were calculated to define whether a disease was considered to be associated with a given cluster. Diseases with an O/E ratio ≥2% or exclusivity ≥25% were identified to be associated with a given cluster. More details on O/E ratios, disease exclusivity and k-means algorithm have been described in previous studies (Guisado-Clavero et al., 2018; Marengoni et al., 2020). Finally, generalized estimating equation (GEE) model with unstructured working correlation matrix was used to evaluate the association between functional impairment and co-occurring DSI and multimorbidity. We adjusted for all covariates including sociodemographic characteristics, lifestyle factors and health conditions when performing a set of multivariable models. For all models, odds ratio (OR) with corresponding 95% confidence interval (95% CI) were reported. A 2-tailed *p* < 0.05 was interpreted as statistically significant. All statistical analyses were performed using Stata 14.2 (StataCorp, College Station, TX, United States).

## Results

Table 1 shows demographic characteristics of participants, stratified by SI and multimorbidity. At baseline, the mean age of participants was 58.52 (SD = 8.64) years and 53.8% of the participants were female. About 5.1% reported HI only, 28.2% reported VI only, 27.9% reported DSI only, and 22.6% reported DSI and multimorbidity. DSI and multimorbidity was more common among those who were female, older, less educated, unmarried, and living in rural areas.

**TABLE 1:** Sample characteristics by sensory impairment and multimorbidity at baseline (*N* = 10,217).

Variables	No SI	HI only	VI only	DSI only	DSI + Multimorbidity	*p*
**Gender**						
Male	869 (18.4)	279 (5.9)	1296 (27.5)	1316 (27.9)	961 (20.4)	**<0.001**
Female	789 (14.4)	242 (4.4)	1589 (28.9)	1532 (27.9)	1344 (24.5)	
**Age (Mean ± SD)**	56.7 ± 8.7	58.9 ± 9.1	57.3 ± 8.0	59.0 ± 8.9	60.6 ± 8.4	**<0.001**
**Education**						**<0.001**
Lower than primary school	421 (12.7)	159 (4.8)	899 (27.1)	1019 (30.6)	830 (24.9)	
Primary school	568 (14.8)	163 (4.2)	1062 (27.6)	1087 (28.2)	970 (25.2)	
Middle school or above	669 (22.0)	199 (6.6)	924 (30.4)	742 (24.4)	505 (16.6)	
**Marital status**						**0.006**
Married or partnered	1506 (16.5)	461 (5.0)	2598 (28.4)	2556 (28.0)	2017 (22.1)	
Unmarried or others	152 (14.1)	60 (5.6)	287 (26.6)	292 (27.1)	288 (26.7)	
**Residence**						**<0.001**
Urban areas	352 (19.9)	99 (5.6)	539 (30.5)	385 (21.8)	392 (22.2)	
Rural areas	1291 (15.4)	418 (5.0)	2326 (27.8)	2445 (29.2)	1901 (22.7)	
**Smoking status**						**0.002**
No	1115 (15.7)	362 (5.1)	1996 (28.1)	1960 (27.6)	1681 (23.6)	
Yes	543 (17.5)	159 (5.1)	889 (28.7)	888 (28.6)	624 (20.1)	
**Alcohol drinking**						**0.006**
No	1069 (15.6)	347 (5.1)	1921 (28.3)	1904 (27.8)	1613 (23.5)	
Yes	589 (17.5)	174 (5.2)	964 (28.7)	944 (28.1)	692 (20.6)	

*Bold values denotes statistical significance (p < 0.05).*

**FIGURE 1:**
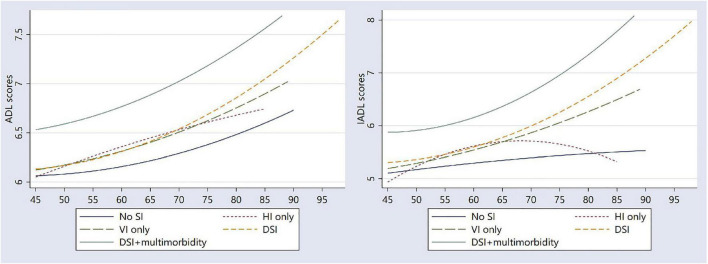
Trajectories of ADL and IADL scores by the presence of sensory impairment and multimorbidity.

Figure 1 shows the trajectories of ADL limitations and IADL limitations across different SIs and multimorbidity groups. The ADL and IADL trajectories among adults with DSI and multimorbidity took an upward curvilinear shape, increasing with age. Adults with DSI showed a faster trajectory of functional decline than those without SI or HI only or VI only.

Of the total 14 chronic disease conditions, 13 conditions with a prevalence of >2% were included in the pattern analysis. Five multimorbidity patterns were identified at baseline: the cardio-cerebrovascular pattern (including hypertension, diabetes, heart diseases, stroke, and cancer; *n* = 795, 20.6%), the stomach-arthritis pattern (including stomach or other digestive disease, arthritis; *n* = 1,756, 45.6%), the respiratory pattern (including chronic lung disease and asthma; *n* = 449, 11.7%), the hepatorenal pattern (kidney disease and liver disease; *n* = 431, 11.2%), and the unspecified pattern (*n* = 423, 11.0%) (Supplementary Table 1).

Table 2 presents results from multivariate logistical analyses that examined the associations between functional impairment and the co-occurrence of SIs and multimorbidity. After controlling for gender, age, education, marital status, residence, smoking status, and alcohol drinking, adults with no SIs were less likely to report ADL limitations (OR = 0.54, 95% CI: 0.48–0.61) and IADL limitations (OR = 0.52, 95% CI: 0.46–0.58) compared with those with DSI. Compared with adults with DSI only, those with DSI and multimorbidity were 127 and 89% more likely to report ADL limitations and IADL limitations, respectively. Compared with adults with DSI only, those with VI only were less likely to suffer from ADL limitations (OR = 0.90, 95% CI: 0.83–0.98) and IADL limitations (OR = 0.88, 95% CI: 0.81–0.95), and those with HI only were less likely to suffer from IADL limitations (OR = 0.79, 95% CI: 0.69–0.91).

**TABLE 2:** Longitudinal associations between functional impairment and the co-occurrence of sensory impairment and multimorbidity (*N* = 10,217).

Model	ADL		IADL	
	OR	95%CI	OR	95%CI
No SI	0.54**	0.48–0.61	0.52**	0.46–0.58
HI only	0.93	0.81–1.08	0.79**	0.69–0.91
VI only	0.90*	0.83–0.98	0.88**	0.81–0.95
DSI only	Ref		ref	
DSI + Multimorbidity	2.27**	2.11–2.43	1.89**	1.77–2.02

*Model was adjusted for gender, age, education, marital status, residence, follow-up year, depressive symptoms, smoking status and alcohol drinking status.*

*p < 0.05 **p < 0.01.

Table 3 shows the results of generalized estimating equation models for the longitudinal relationships between subsequent functional impairment and DSI and the multimorbidity patterns at baseline. The analysis was restricted to those with DSI. After adjusting for potential confounding variables, all occurrence of DSI and multimorbidity patterns presented statistically significant associations with ADL limitations compared with DSI only, with odds ratios ranging from 1.66 (95% CI: 1.39–1.99) for DSI and the unspecific pattern to 2.70 (95% CI: 2.34–3.12) for DSI and the hepatorenal pattern. In the fully adjusted longitudinal analyses, all occurrence of DSI and multimorbidity patterns were associated with a higher risk of having IADL limitations, with odds ratios ranging from 1.61 (95% CI: 1.42–1.83) for DSI and the cardio-cerebrovascular pattern to 2.04 (95% CI: 1.77–2.36) for DSI and the hepatorenal cluster.

**TABLE 3:** Longitudinal association between functional impairment and DSI and multimorbidity patterns (*N* = 5,153).

Model	ADL		IADL	
	OR	95%CI	OR	95%CI
DSI	Ref		Ref	
DSI + the cardio-cerebrovascular pattern	1.85**	1.63–2.11	1.61**	1.42–1.83
DSI + the stomach-arthritis pattern	1.91**	1.75–2.09	1.62**	1.48–1.76
DSI + the respiratory pattern	2.11**	1.82–2.44	1.99**	1.72–2.30
DSI + the hepatorenal pattern	2.70**	2.34–3.12	2.04**	1.77–2.36
DSI + the unspecified pattern	1.66**	1.39–1.99	1.81**	1.53–2.15

*Model was adjusted for gender, age, education, marital status, residence, follow-up year, depressive symptoms, smoking status and alcohol drinking status.*

*p < 0.05 **p < 0.01.

## Discussion

Using nationally representative data, this study attempts to improve our understanding of the relationship between DSI and its interactive effect with multimorbidity on functional impairment among middle-aged and older adults in China. First, DSI is strongly associated with functional impairment including ADL and IADL limitations. Second, the co-occurrence of DSI and multimorbidity is associated with a more-than-doubled elevated increased risk of functional impairment. Preventing functional impairment is easier to succeed than attempting to regain lost autonomy (Rodriguez-Manas and Rodriguez-Sanchez, 2021), therefore, screening people with DSI and multimorbidity is vital to identify adults with functional impairment in future.

### The Effect of Dual Sensory Impairment on Functional Impairment

Similar to previous studies (Guthrie et al., 2016; Bouscaren et al., 2019; Davidson and Guthrie, 2019), the current study found that adults with DSI were more likely to lose independence in ADL and IADL. A plausible channel linking DSI with functional impairment was through depression or social isolation. DSI has been found associated with depression and social isolation (Hajek and Konig, 2020; Xie et al., 2021), both of which are important contributors to functional impairment. Factors related to DSI, such as physical balance and fear of falls, have also been discussed as underlying mechanisms for the relationship between DSI and functional impairment (van Landingham et al., 2012). Our study also found individuals with DSI were at compounded risk of IADL limitations. One possible reason was that vision and hearing tend to affect different IADL domains. Successful completion of IADL may be more challenging when both hearing and vision were impaired, while single SI could be more likely compensated (Davidson and Guthrie, 2019).

### The Combined Effect of Dual Sensory Impairment and Multimorbidity on Functional Impairment

Based on the finding of the relationship between DSI and functional impairment, our study further found a combined effect of DSI and multimorbidity on ADL and IADL limitations. This novel finding demonstrates the additive effect of multimorbidity on functional impairment among adults with DSI. As shown in Table 1, the DSI + multimorbidity group was more likely less educated, unmarried, and living in rural areas. These socioeconomic factors could be associated with less healthy lifestyles and poorer chronic disease care and thus contribute to worse physical function (Song et al., 2017; Luo et al., 2020). Previous studies have demonstrated that each additional chronic condition was associated with an increasing level of functional impairment (Jindai et al., 2016; Quinones et al., 2016; Bowling et al., 2019), thus multimorbidity worked with DSI to further increase the likelihood of functional impairment. This result indicates that integrated services may be particularly valuable for adults with combinations of DSI and multimorbidity. Moreover, symptom severity was associated with the development of functional impairment and may be a mediating variable between multimorbidity and functional impairment (Portz et al., 2017). Future research should consider the severity of chronic disease and validate it. Additionally, some previous studies regard SI as a chronic disease to explore the effects of multimorbidity patterns on functional impairment (Marengoni et al., 2020, 2021). However, doing so may mask the combined effect of DSI and other chronic diseases on functional impairment. This study provides new insight for disability prevention by exploring the combined effect of DSI and multimorbidity patterns on functional impairment exactly.

This study found that co-occurrence of DSI and the hepatorenal pattern was most strongly associated with ADL and IADL limitations. One possible reason was that DSI and the hepatorenal diseases affect functional status through different mechanisms. Chronic diseases that affect functional status through different mechanisms appear to have a worse effect on functional impairment than those share aetiologic factors or pathophysiologic mechanism (Kriegsman et al., 2004). Different from DSI, kidney disease and liver disease may lead to malnutrition, inflammatory and sarcopenia, which are key components of functional impairment (Fried et al., 2006; Samoylova et al., 2017). Simultaneously, this study found that baseline presence of DSI and the cardio-cerebrovascular pattern could predict functional impairment during follow-up period, but its effect size is a little lower than other groups. Previous studies have demonstrated that hypertension, diabetes, heart diseases, and stroke in the cardio-cerebrovascular pattern increase the risk of VI (Garin et al., 2014; Zheng et al., 2020), which might explain the higher frequencies of co-occurrence of DSI and the cardio-cerebrovascular pattern and the weaker effect of this co-occurrence on functional impairment. The groups of DSI and other multimorbidity patterns demonstrated significant effects on functional impairment. These findings pointed out the complexity and heterogeneity of multimorbidity and its cumulative effect with DSI for middle-aged and older adults functional status, suggesting the need to develop tailored interventions for them.

### The Effect of Hearing Impairment on Functional Impairment

Many studies have examined the relationship between HI and functional impairment, but the results were inconsistent. Some studies presented an insignificant association between HI and functional impairment (Rudberg et al., 1993; Lin et al., 2004; Brennan et al., 2006). However, our study found HI was strongly associated with functional impairment, which was consistent with results of some studies (Tareque et al., 2019; Martinez-Amezcua et al., 2021). Interestingly, these studies that find no association relied on data collected before age 30 and thus might not represent the current generation of older adults. More recent conceptualizations of the disablement process indicate that impairments may lead to ADL/IADL limitations and participation restrictions (Xiang et al., 2020). Our study based on data from 2011 to 2018 demonstrated not only a substantial association between HI only and functional impairment, but the association between DSI and ADL limitations that was driven primarily by HI. Thus, it is in urgent need to conduct treatment measures for middle-aged and older adults with HI.

#### Implications

Although SI is common in older adults, approximately 88% of VI can be prevented and treated by wearing glasses and receiving cataract surgery (World Health Organization, 2013), and HI can be treated effectively by using hearing aids and other assistive devices (World Health Organization, 2020). Treatments for cataract and refractive error are highly cost-effective and would meet more than 90% of unmet needs and further improve general and mental health as well as health equity (Burton et al., 2021). However, there is an 83% unmet need for hearing aids globally (Orji et al., 2020). Heine et al. (Heine et al., 2019) reported that only 0.8% of older adults wore hearing aids, although the proportion with HI was high (64.9%) in China. Our study implied that health policy makers should develop applicable approaches to promote the use of assistive equipment (glasses or hearing aids) and therefore minimize the impact of DSI on functional ability. In addition to glasses or hearing aids, rehabilitation therapy services (training of remaining/residual senses or environmental adaptations) could also help people with DSI regain their functional independence (Jaiswal et al., 2018). Individuals with DSI have different support needs due to different levels of support in their environment, the severity of DSI, and the age of onset (World Federation of the Deafblind, 2018). For older adults with DSI, those experience different multimorbidity patterns may have more additional support needs. Therefore, clinicians could provide more precise treatment and management plans for adults with DSI based on their multimorbidity patterns to prevent or delay functional impairment.

Our study also has several limitations. Firstly, SI was assessed by self-reported measures in this study. On the one hand, self-reported sensory impairment is valuable in capturing people’s perception of their functional hearing and vision and suitable for illustrating the meaning of DSI for daily life (Assi et al., 2020; Hajek and Konig, 2020). On the other hand, self-reported impairments may be subject to bias because factors such as cognitive status, education, age, and/or individual disposition may contribute to the determination of self-reported impairments. Therefore, future study is suggested to examine these associations by using objective measurements. Secondly, the severity and duration of SI and multimorbidity were not measured in this study, which would be further studied in the future. Finally, given the dynamic nature of multimorbidity, adults in the unspecific pattern would have changed to a pattern in the follow-up waves of survey.

## Conclusion

In this prospective study, DSI was significantly associated with ADL and IADL limitations in middle-aged and older adults. Middle-aged and older adults with DSI, particularly those with co-occurrence of multimorbidity, were at increased risk for functional impairment. Efforts to improve functional status could begin in preventing and treating DSI and managing multimorbidity among middle-aged adults. Middle-aged and older adults with DSI and the hepatorenal pattern represented a high-risk population that could be a target for intervention to prevent functional impairment and promote health aging.

## Data Availability Statement

The datasets used in this study are available at: http://charls.pku.edu.cn/index/en.html.

## Ethics Statement

The ethical review committee of Peking University approved CHARLS and informed consents are signed by participants before their participation. This study was reviewed and approved by Peking University’s Institutional Review Board, IRB00001052-11015. The patients/participants provided their written informed consent to participate in this study.

## Author Contributions

CZ and QW: conceptualization. QW, SZ, YW, and DZ: formal analysis. QW: drafting of the manuscript. CZ, XC, and QW: critical revision. All authors approved the final version to be published.

## Conflict of Interest

The authors declare that the research was conducted in the absence of any commercial or financial relationships that could be construed as a potential conflict of interest.

## Publisher’s Note

All claims expressed in this article are solely those of the authors and do not necessarily represent those of their affiliated organizations, or those of the publisher, the editors and the reviewers. Any product that may be evaluated in this article, or claim that may be made by its manufacturer, is not guaranteed or endorsed by the publisher.
