# Supplementary material for: The Effect of Dual Sensory Impairment and Multimorbidity Patterns on Functional Impairment: A Longitudinal Cohort of Middle-Aged and Older Adults in China
Source: Front Aging Neurosci. 2022 Apr 8;14:807383. doi: 10.3389/fnagi.2022.807383 (PMC9028763; doi:10.3389/fnagi.2022.807383)
Supplement: Supplementary file 1 [file Table_1.DOCX]

Table S1: Prevalence, exclusivity, and observed/expected ratio of diseases in multimorbidity patterns

| Disease | Prevalence within pattern (%) | Exclusivity (%) | Observed/Expected Ratio |
| --- | --- | --- | --- |
| hypertension | 89.3 | 46.4 | 1.9 |
| diabetes or high blood sugar | 60.3 | 21.5 | 2.8 |
| cancer | 32.1 | 12.3 | 2.6 |
| chronic lung disease | 99.3 | 21.3 | 4.7 |
| liver disease | 13.0 | 9.0 | 1.5 |
| heart disease | 45.8 | 27.3 | 1.7 |
| stroke | 9.3 | 4.5 | 2.1 |
| kidney disease | 100.0 | 14.4 | 7.1 |
| stomach or other digestive disease | 98.3 | 47.0 | 2.1 |
| emotional, nervous, or psychiatric disease | 2.63 | 2.5 | 1.1 |
| memory-related disease | 4.3 | 2.6 | 1.7 |
| arthritis or rheumatism | 9.4 | 64.8 | 1.5 |
| asthma | 30.5 | 7.9 | 3.9 |
